# Endoscopic Treatment and Pulmonary Rehabilitation for Management of Lung Abscess in Elderly Lymphoma Patients

**DOI:** 10.3390/ijerph17030997

**Published:** 2020-02-05

**Authors:** Roberto Cascone, Antonello Sica, Caterina Sagnelli, Annalisa Carlucci, Armando Calogero, Mario Santini, Alfonso Fiorelli

**Affiliations:** 1 Department of Translation Medicine, Thoracic Surgery Unit, University of Campania “Luigi Vanvitelli”, 80131 Naples, Italy; rob.cascone@libero.it (R.C.); annalisacarlucci88@gmail.com (A.C.);; 2 Department of Precision Medicine, University of Campania Luigi Vanvitelli, 80131 Naples, Italy; antonellosica@gmail.com; 3 Department of Mental Health and Public Medicine, University of Campania Luigi Vanvitelli, 80131 Naples, Italy; caterina.sagnelli@unicampania.it; 4 Department of Advanced Biomedical Sciences, University of Naples Federico II, 80131 Naples, Italy; armando.calogero2@unina.it

**Keywords:** lung abscess, endoscopy, pulmonary rehabilitation, elderly lymphoma patients

## Abstract

*Background*: The management of lung abscess may be a challenge in elderly patients undergoing chemotherapy and/or radiotherapy for previous malignancy. Herein, we reported a case series of elderly patients with previous lymphoma undergoing endoscopic treatment followed by pulmonary rehabilitation for lung abscess. *Methods*: Our study population included a consecutive series of elderly patients with previous lymphoma and lung abscess. Suppurative infection was refractory with specific antibiotic therapy. In all cases, drainage was endoscopically inserted in lung abscess via video-bronchoscopy. This strategy allowed performing daily therapy with the installation of gentamicin directly into the abscess cavity. All patients underwent a respiratory rehabilitation program to speed up convalescence and allow early discharge. *Results*: After positioning the catheter through a bronchoscopic route and subsequent washing with gentamicin, all the patients in our study showed an improvement in clinical conditions with resolution of fever within a few days of starting the procedure with normalization of blood tests (mean hospital length 7 ± 0.73 days). A follow-up chest computed tomography scan showed a resolution of lung abscess within a mean of 27 ± 1.53 days. *Conclusions:* Endoscopic treatment with a rehabilitation program may be a valuable strategy for the management of lung abscess that is refractory to standard antibiotic therapy. Further and larger studiesshould be done to confirm our results.

## 1. Introduction

Lung abscess is an infectious disease that often does not respond to antibiotic treatment, requiring invasive therapies such as the placement of percutaneous drainage. The suppurative process can be a primary event and a direct consequence of the aspiration of alimentary material, of necrotizing pneumonia or caused by states of immunodepression (e.g., steroid therapy, chemotherapy, immunosuppression in transplanted patients) [1,2,3,4,5,6,7].

The clinical manifestations of lung abscess cover a very wide spectrum, ranging from simple coughing to a full-blown septic picture. The clinical presentation of the infection is varied and depends on multiple factors such as the pathogen involved, the stage of the disease in which the patient is undergoing medical evaluation, and intrinsic factors of the subject, such as the normal function of the immune system. The symptoms of lung abscess can develop following pneumonia after a few weeks, appearing lighter than those of the latter: the fever is generally not high and the patientsreport significant weight loss, as well as the presence of productive cough, often with foul-smelling sputum, and fatigue. The prognosis essentially depends on the presence of respiratory, cardiac and, above all, neurological comorbidities. In the latter category of patients, due to neurological diseases that alter normal esophageal function, the persistence or the recurrence of the lung abscess is frequent due to continuous aspirations of ingested material.

The management of lung abscess may be challengingin elderly patients, even more so in those undergoing chemotherapy and/or radiotherapy for previous malignancy. The elderly patient can present a series of neurological pathologies (dementia, stroke, neuromuscular diseases) which increase the risk of aspiration of ingested material and, consequently, of aspiration pneumonia. Furthermore, compulsory stay in hospitals or nursing homes, with the consequent nosocomial infections, affects the already precarious immune system of these subjects. The result is an ineffectiveness or a failure of antibiotic therapy which results in a severe prognosis. From this problem arises the need to identify an effective treatment to improve the eradication of the infectious process in the elderly patient, without exposing it to the risks associated with invasive surgical treatment.

Herein, we reported a case series of elderly patients with previous lymphoma undergoing endoscopic treatment followed by active lung physiotherapy for lung abscess.

## 2. Materialsand Methods

Our study population included 12 consecutive elderly patients (mean age, year 69 ± 2.6) with lung abscess and previous lymphoma. The characteristics of the study population are summarized in Table 1. At the time of admission, patients presented with fever, malaise, cough with foul sputum, and weight loss. Patients were also tested for HBsAg, anti-HCV, total anti-HBc, and anti-hepatitis B surface antibody (HBs) using specific commercial immunoenzymatic assays as described in previous studies [8,9,10,11,12]. Performing a CT scan confirmed the diagnosis of abscess due to the presence of a cavitated lung lesion, with a hydro-air level. Through flexible bronchoscopy, bronchoalveolar lavage and a culture test was performed to identify the pathogen responsible for the lung abscess.Once the pathogen was isolated, as reported in Table 1, an antibiogram was performed to select the best antibiotic for the treatment of the infection.

In all 12 cases, a histological examination was performed on samples obtained with transbronchial biopsy, in order to exclude a possible relapse of disease masked by a pulmonary abscess. In addition, in 4 patients who showed suspicious images at the CT scan of lung cancer with suppuration or a relapse of lymphoproliferative disease within the lung, a transparietal fine-needle aspiration cytology (FNAC) and a cytological examination were performed. Finally, to obtain further confirmation of no relapse, bronchoalveolar lavage and a subsequent cytological examination of the sample were performed in all patients.

In all patients, antibiotic therapy was ineffective: the clinical, radiological and laboratory findings were unchanged in 10 patients while in two patients there was an increase in the size of the abscess cavity as well as the persistence of fever and leukocytosis. Before proceeding with the endoscopic treatment, an arterial blood gas test and a spirometry were performed to assess the degree of oxygenation and lung function respectively. All patients analyzed in our study had a mild degree of hypoxemia, without pCO2 and pH alterations. In all the patients, spirometry showed a reduction in forced expiratory volume in one second (FEV1%)(mean value 68 ± 5.2) and in forced vital capacity (FVC%) values(mean value 71% ± 4.8%).

### 2.1. Endoscopic Procedure

**Figure 1:**
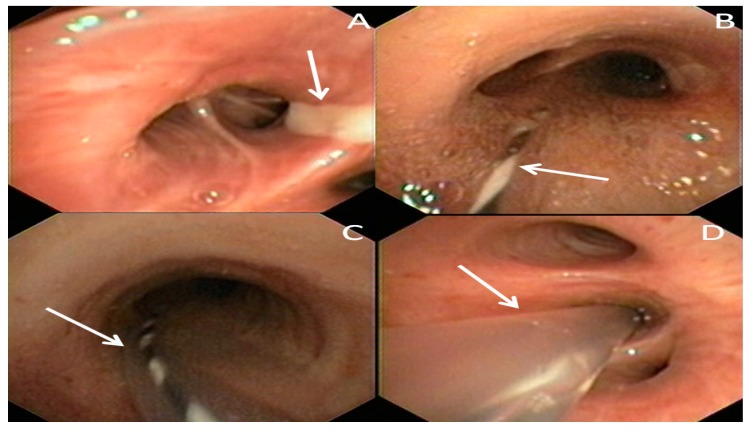
Video-bronchoscopy identified the bronchial segments site of the lung abscess cavity (white arrow) (**A**). A guide-wire (white arrow) was then inserted within the affected bronchial segment (**B**). Then, a catheter is inserted over the guidewire (white arrow) (**C**), and placed within the affected bronchial segment (white arrow) (**D**).

**Figure 2:**
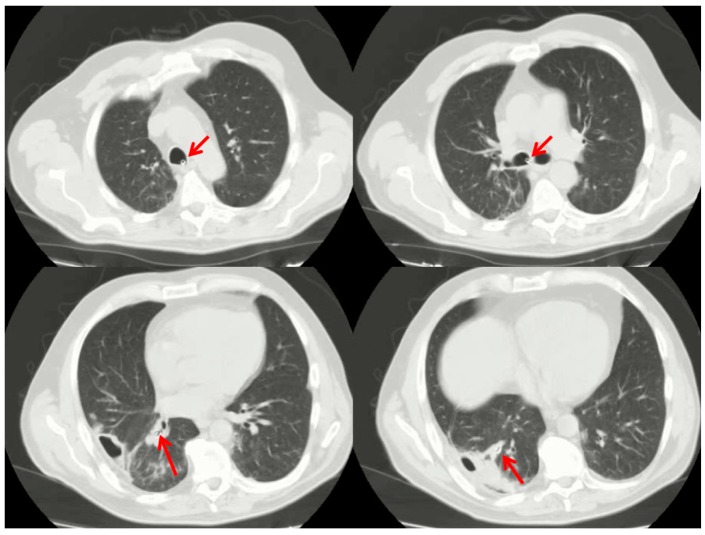
Chest computed tomography scan confirmed the prompt position of catheter (red arrows).

The procedure is summarized in Figure 1. It was performed in a dedicated endoscopic room under moderate sedation and spontaneous ventilation. Video-bronchoscopy identified the bronchial segments site of the lung abscess cavity (Figure 1A) and a guide-wire was then inserted through the operator channel. Once the correct positioning of the guide-wire was established (Figure 1B), the bronchoscope was carefully removed, and a small catheter was inserted over the guidewire (Figure 1C) within the bronchial segment site of the abscess (Figure 1D). This catheter was then fixed externally to a nasal level to prevent accidental extraction. Through it, it was possible to perform daily therapy with the instillation of gentamicin directly into the abscess cavity. In addition to local therapy, patients also continued treatment with systemic antibiotics. Furthermore, samples could be taken daily for culture, allowing us to calibrate the most effective antibiotic therapy from time to time. The catheter was removed after the complete resolution of the clinical findings and after normalization of white blood cells. Then, a chest computed tomography scan was performed to check the prompt position of the catheter (Figure 2).

### 2.2. Respiratory RehabilitationProgram

During hospitalization, each patient undertook an enhanced recovery pathway, aimed at speeding up convalescence and allowing early discharge. Among the measures implemented, the most important was respiratory rehabilitation: this was made possible also thanks to the simple management of the catheter and its poor interference on the exercises performed by the patient. Patients were followed by a physiotherapist who practiced two sessions of chest physiotherapy per day: the exercises included breathing techniques and specific positioning to promote adequate lung expansion, and exercises to improve the airway clearance, using incentive spirometers. In addition, anaerobic exercises for upper and lower limbs were carried out, as well as walking. This allowed both to improve the complications directly caused by excessive bed rest and to improve the psychological aspect of the patients, thanks to an active solicitation. Following catheter removal and discharge, patients were followed up by the physiotherapist on an outpatient basis for at least 4 weeks, carrying out gradual pulmonary rehabilitation.Another aspect treated in this rehabilitation process was the evaluation of nutritional status, by monitoring the body mass index (BMI) [13], serum albumin, and the use of Nutritional Risk Screening (NRS-2002).

In cases of evident malnutrition, also due to the infectious process, oral supplementation was carried out or, in the most severe cases, parenteral nutrition was administered.

## 3. Results

No complications during and after the placement of the catheter was observed. After positioning the catheter through a bronchoscopic route and subsequent washing with gentamicin, all the patients in our study showed an improvement in clinical conditions with resolution of fever within a few days of starting the procedure with normalization of blood tests (mean hospital length 10.6 ± 2.2 days). A daily sampling of the infected material through the catheter allowed serialized culture tests with antibiograms to be carried out, allowing antibiotic therapy to be modified in the case of selection of drug-resistant pathogens. In 4 patients, the culture test was negative and therefore continued with broad-spectrum antibiotic therapy.

The cytological examination performed on both BAL and FNAC excluded the relapse of hematological disease or the onset of primary lung cancer in all patients.

**Figure 3:**
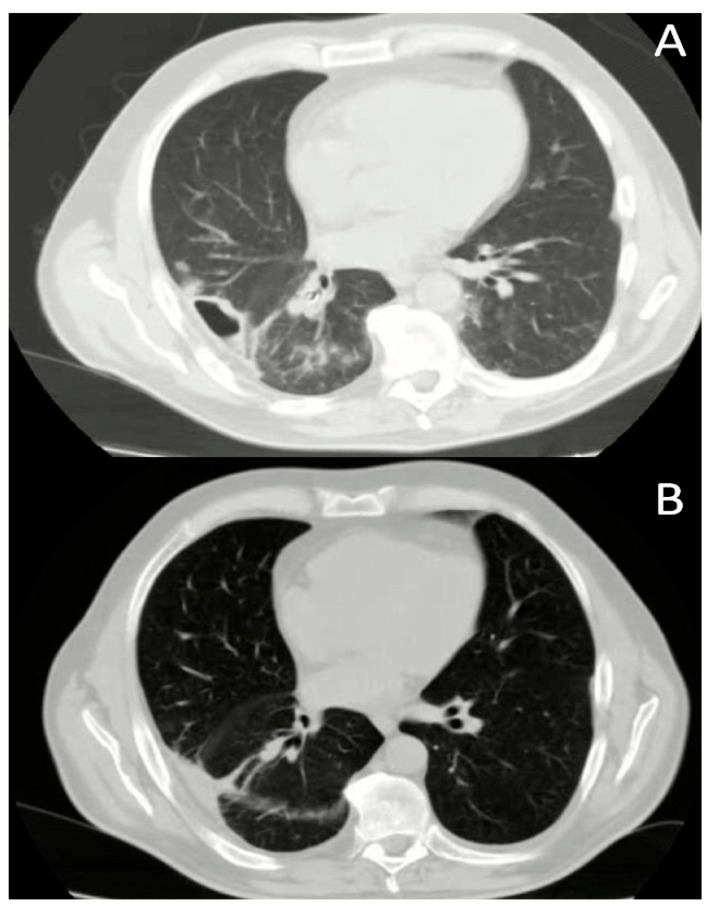
Chest computed tomography scan before (**A**) and after treatment (**B**).

A follow-up chest computed tomography scan showed a resolution of lung abscess within a mean of 27 ± 1.53 days. An exampleisreported in Figure 3. Although all the subjects showed a remission of clinical symptoms following endoscopic treatment, only a partial reduction of the abscess cave was found in 2 patients on CT scan. However, at the outpatient examination after twomonths from discharge, both showed on CT scan a complete obliteration of the cave and the consequent formation of a scar in the lung parenchyma.

Spirometries performed at discharge and after 4 weeks of pulmonary rehabilitation showed a significant improvement in lung function. The comparison between the pre-procedural data and the data of the last follow-up after rehabilitation showed a significant increment of FEV1% (68% ± 5.2% versus 82% ± 4.3%; *p* = 0.003, Student *t*-test)and of FVC% (71% ± 4.8% versus 83% ± 8.3; *p* = 0.002, Student *t*-test).

## 4. Discussion

Lung abscess is an infectious disease that can normally be treated with conservative therapy.

The first choice is to set up a broad-spectrum antibiotic therapy, waiting to isolate the pathogen responsible for suppuration by culturing bronchoalveolar lavage.From the results obtained in our study, one of the advantages that we want to underline is the possibility of obtaining, through the catheter positioned endoscopically, repeated samples of the infectious material without the need to perform additional bronchoscopies. This allows us, on the one hand, to carry out a continuous clearance of the abscess cave and, on the other, to carry out serial antibiograms on the pathogen that is isolated from the culture test. Thanks to this, antibiotic therapy can be readily changed if a pathogen with antibiotic resistance is to be isolated. In patients with particular comorbidities, such as immunosuppression, medical therapy alone may not be sufficient to completely eradicate the infectious process: therefore, invasive methods are applied to obtain a good abscess cavern clearance [14].

The use of percutaneous abscess drainage was first described in 1938 by Neuthof et al. [15] and, subsequently, deepened by Monaldi et al. [16] for the treatment of the tubercular cavern.

In the pre-antibiotic era, this method was for a long time the first choice for the treatment of the suppurative processes of the lung: to date, it remains the best choice in case of failure of standard antibiotic therapy in 11%–20% of patients with lung abscess [1].

In the event that the infectious process involves a large part of the lung lobe and when the prospect of complete recovery is uncertain, an anatomical resection of the affected lobe is mandatory.

The failure of antibiotic therapy can be the result of various components, for example, an inadequate function of the immune system.

This occurs mostly in patients suffering from a congenital or acquired immunodeficiency (e.g., AIDS) or in subjects undergoing organ transplantation and in therapy with immunosuppressive drugs [17].

From the analysis of patients with lung abscess who came to our attention, a non-negligible proportion had a clinical history of a lymphoproliferative process previously treated with chemotherapy but, at the time of admission, with no finding of disease [18].

One of the problems we must face most of these patients is the differential diagnosis between primary lung abscess and lymphoma with pulmonary localizationand concomitant bacterial suppuration.

In fact, although rare, it is not impossible to find a lymphoma at the level of the lung with totally aspecific clinical and radiological characteristics and that incorrectly direct towards a diagnosis of abscess [19].

It is therefore essential to obtain an adequate sampling of the bronchoscopy material to perform a histological examination in addition to the cultural one. The direct sampling carried out with BAL through the catheter positioned endoscopically allowed us to exclude through the cytological examination a relapse of lymphoproliferative disease or the onset of a primary lung cancer.

Although with low sensitivity, the cytological examination of the sputum may be useful and easy to perform, which can orient early towards the suspicion of primitive lymphoma [20].

As reported by Yuba et al. [10], a further problem of difficult management is the reactivation of chronic infectious processes, such as tuberculosis, due to the chemotherapy treatment of lymphoma.

In fact, the antineoplastic drugs commonly used for the treatment of lymphoproliferative processes alter the normal function of the immune system, causing the reactivation of pre-existing infections.

In such cases, the advantages and disadvantages of stopping chemotherapy should be weighed to allow adequate treatment of the existing infective process [21,22,23].

In all patients with existing lymphoproliferative disease or already undergoing chemotherapy, we hypothesize that this haematological pathology correlates with a dysfunction of the immune system, increasing the risk of developing a lung abscess compared to patients without lymphoma.

Furthermore, this causes a lower susceptibility to standard antibiotic treatment and the consequent therapeutic failure, making it necessary to place a drainage of the abscess cavity.

In our experience, the positioning of an endoscopic catheter allows us to better control the infectious outbreak in these patients at risk, performing a daily clearance of the lesion and allowing us to carry out more sampling without the need for further bronchoscopy, also improving the compliance of the patients.

The use of fibrinolytics has been described for the first time by Haaga et al. in 1988 but used essentially for the treatment of the multi-locus pleural empyema; in the literature, the data on the use of these drugs for abscesses derive mostly from studies on abdominal abscesses [24].

A noteworthy work is that of Chae et al. [25] which described the use of fibrinolytic drugs in the treatment of a lung abscess in a patient with large B cell lymphoma.

One of the adverse effects that most alarms us is, however, the increased bleeding risk caused by the fibrinolytic activity which, added to the haematological pathology, is in our opinion a sufficient reason to advise against or at least accurately assess the use of these drugs.

In the management of our infectious patients, we have chosen to apply some elements deriving from the Enhanced Recovery After Surgery (ERAS) protocols: these included a respiratory rehabilitation after catheter positioning, monitoring and treatment of malnutrition states and anemia, continuous mobilization to reduce the thromboembolic risk, and pain management with specific analgesic techniques for each patient [26].

This has allowed us to improve the convalescence, allow an early discharge to the home once the infectious picture has been resolved from the clinical and laboratory point of view, thus reducing the complications typical of a long period of hospital stay (e.g., further infections, venous thromboembolism for excessive bed rest, etc.).

The fundamental point that we would like to underline in our work is the need for chest physiotherapy performed early during hospitalization and continuation of pulmonary rehabilitation up to 4 weeks after discharge. Usually, the elderly patient has numerous comorbidities of which the most frequent is COPD [27,28,29,30]. This pathology, associated with the destruction of the lung parenchyma by the abscess, affects the respiratory performance and, consequently, the quality of life of the subject. As evidenced by our results, the respiratory function of the patients analyzed with the execution of a spirometry in the outpatient clinic was significantly improved. The presence of the catheter is not a contraindication to performing the exercises during hospitalization, as our patients have completed all physiotherapy sessions with good compliance.

## 5. Conclusions

The lungabscessin the elderlypatient, concomitant or subsequent a lymphoma, is not a rare event and it requires special attention in its management. Among these, there is the concrete possibility of failure of antibiotic therapy or the need for continuous drainage of infectious samples for the execution of serial antibiograms. In our experience, we believe that bronchoscopic positioning of a catheter fixed externally at a nasal level for the continuous drainage of purulent material and continuous medication allows a faster resolution of the infectious process, especially in those patients susceptible to this disease like those with lymphoma. Although the complete radiological resolution of the abscess cave may occur in some cases after a long time, our procedure still allows to contrast the septic picture and improve the clinical setting, a primary point in the management of a complicated patient such as the elderly person with a history of neoplastic disease.Furthermore, the application of methods deriving from the ERAS pathways allows us to speed up the full recovery of the patient with lung abscess and to enable a rapid discharge to the home.

## Figures and Tables

**Table 1:** The characteristics of 12 elderly patients with lung abscess and previous lymphoma.

Patient No.	Lymphoproliferative Disease	Sex	Age, Years	Catheter, Days	Cultural Test	Discharge, Days
1	Hodgkinlymphoma	M	54	3	*Staphylococcus aureus*	7
2	Large B-cell lymphoma	M	67	4	*Enterobacter* sp.	12
3	Follicular lymphoma	F	49	4	negative	6
4	Hodgkin lymphoma	M	72	3	*Pseudomonas* sp.	8
5	Large B-cell lymphoma	F	70	5	*Staphylococcus aureus*	8
6	Hodgkin lymphoma	M	64	4	Negative	10
7	Mycosisfungoides	F	55	3	*Klebsiella* sp.	9
8	Large B-cell lymphoma	F	58	4	*Enterobacter* sp.	7
9	Hodgkin lymphoma	M	61	4	negative	8
10	Hodgkin lymphoma	M	45	5	*Klebsiella* sp.	13
11	MALT lymphoma	M	53	4	negative	15
12	Large B-cell lymphoma	F	72	5	*Enterobacter* sp.	9
